# GeneBreak: detection of recurrent DNA copy number aberration-associated chromosomal breakpoints within genes

**DOI:** 10.12688/f1000research.9259.2

**Published:** 2017-07-06

**Authors:** Evert van den Broek, Stef van Lieshout, Christian Rausch, Bauke Ylstra, Mark A. van de Wiel, Gerrit A. Meijer, Remond J.A. Fijneman, Sanne Abeln

**Affiliations:** 1 Department of Pathology, VU University Medical Center, Amsterdam, 1081 HZ, Netherlands; 2 Department of Epidemiology & Biostatistics, VU University Medical Center, Amsterdam, 1081 HZ, Netherlands; 3 Department of Mathematics, VU University Medical Center, Amsterdam, Amsterdam, 1081 HV, Netherlands; 4 Department of Computer Science, VU University Medical Center, Amsterdam, 1081 HV, Netherlands; 5 Department of Pathology, Netherlands Cancer Institute, Amsterdam, 1066CX, Netherlands

**Keywords:** structural chromosomal aberrations, recurrent breakpoint genes, molecular characterization, cancer genome, copy number aberration profile, computational method

## Abstract

Development of cancer is driven by somatic alterations, including numerical and structural chromosomal aberrations. Currently, several computational methods are available and are widely applied to detect numerical copy number aberrations (CNAs) of chromosomal segments in tumor genomes. However, there is lack of computational methods that systematically detect structural chromosomal aberrations by virtue of the genomic location of CNA-associated chromosomal breaks and identify genes that appear non-randomly affected by chromosomal breakpoints across (large) series of tumor samples. ‘GeneBreak’ is developed to systematically identify genes recurrently affected by the genomic location of chromosomal CNA-associated breaks by a genome-wide approach, which can be applied to DNA copy number data obtained by array-Comparative Genomic Hybridization (CGH) or by (low-pass) whole genome sequencing (WGS). First, ‘GeneBreak’ collects the genomic locations of chromosomal CNA-associated breaks that were previously pinpointed by the segmentation algorithm that was applied to obtain CNA profiles. Next, a tailored annotation approach for breakpoint-to-gene mapping is implemented. Finally, dedicated cohort-based statistics is incorporated with correction for covariates that influence the probability to be a breakpoint gene. In addition, multiple testing correction is integrated to reveal recurrent breakpoint events. This easy-to-use algorithm, ‘GeneBreak’, is implemented in R ( *www.cran.r-project.org*) and is available from Bioconductor ( *www.bioconductor.org/packages/release/bioc/html/GeneBreak.html*).

## Introduction

Tumor development is driven by irreversible somatic genomic aberrations such as single nucleotide variants (SNVs) and chromosomal aberrations including numerical as well as structural changes ^1, 2^. Genome-wide somatic DNA copy number aberrations (CNA) profiling is a widely established approach to characterize chromosomal aberrations in cancer genomes. At present, application of computational methods has mainly been focused on the analysis of numerical aberrations of chromosomal segments. Evidence is emerging that genes affected by structural chromosomal aberrations, *i.e.* genes affected by chromosomal breaks, represent a biologically and clinically relevant class of mutations in many cancer types including solid tumors ^3– 6^. Importantly, the actual locations of chromosomal CNA-associated breakpoints, which are the points of copy number level shift in somatic CNA profiles, indicate underlying chromosomal breaks and thereby genomic locations affected by somatic structural aberrations ^5– 12^. Hence, the wide availability of large series of high-resolution DNA copy number data by for instance array-Comparative Genomic Hybridization (CGH) or by (low-pass) whole genome sequencing (WGS) approaches enables to systematically search for regions and genes that are affected by CNA-associated structural chromosomal changes. Computational methods determining numerical CNAs, consequently, also yield CNA-associated breakpoint locations. However, it is not trivial to identify genes that are recurrently affected by CNA-associated chromosomal breakpoints across (large) series of cancer samples since this methodology also requires dedicated computational methods including comprehensive statistical evaluation.

**Figure 1.**
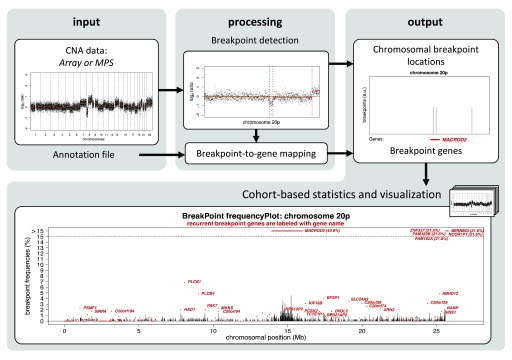
Schematic overview of computational methods. GeneBreak’ requires already segmented DNA copy number data from array-CGH or WGS approaches. The first step involves detection of breakpoint locations. Next, breakpoint locations will be mapped to gene annotations in order to identify genes affected by DNA breakpoints. The final step performs comprehensive cohort-based statistical analyses including correction for multiple testing to reveal both recurrent breakpoint locations and breakpoint genes. The breakpoint frequencies can be visualized with a built-in plot function. This example visualizes the breakpoint locations (vertical black bars) and breakpoint genes (horizontal red bars) on the p-arm of chromosome 20 identified in a cohort of 352 advanced colorectal cancers. The genes labeled with a name are statistically significant recurrent breakpoint genes (FDR<0.1).

We here provide a computational method, ‘GeneBreak’, that identifies chromosomal breakpoint locations using DNA copy number profiles. A tailored annotation approach maps breakpoint locations to genes for each individual profile. Moreover, dedicated comprehensive cohort-based statistical analysis including correction for covariates that influence the probability to be a breakpoint gene and multiple testing pinpoints genes that are non-randomly and recurrently affected by chromosomal breaks across multiple tumor samples ^5^. ‘GeneBreak’ is implemented in R ( www.cran.r-project.org) and is available from Bioconductor ( www.bioconductor.org/packages/release/bioc/html/GeneBreak.html). The Bioconductor vignette describes a detailed example workflow of CNA data obtained by analysis of 200 array-CGH samples. A schematic overview of computational methods is depicted in Figure 1.

## Methods

### DNA copy number profiles

The breakpoint detection method we provide is amenable for data from any DNA copy number discovery platform, *e.g.* array-CGH and (low-pass) WGS. For optimal results, ‘GeneBreak’ takes DNA copy number data that are pre-processed by the R-package ‘CGHcall’ ^13^ or ‘QDNAseq’ ^14^, both based on the Circular Binary Segmentation algorithm ^15^, as input. Alternatively, segmented values (log2-ratios) from a different copy number detection algorithm can be used. In addition, it is recommended to provide discrete DNA copy number states ( *e.g.* loss, neutral, gain) that can be used for breakpoint selection. Bioconductor vignette and manual describe commands and workflows in detail (See Supplementary material).

### Breakpoint detection and filter options

Breakpoints are defined by the chromosomal locations that separate the contiguous DNA copy number segments pinpointed by a segmentation algorithm. ‘GeneBreak’ identifies chromosomal breakpoint locations for each individual DNA copy number profile. Instead of taking all detected breakpoints, users may want to define more precisely what breakpoints to take into account, based on the two flanking DNA copy number segment characteristics. One of the following three selection options can be applied. A) *Copy number-deviation*: this selects breakpoints where the shift in log2-ratio between two consecutive DNA copy number segments exceeds the user-defined threshold; B) *CNA-associated breakpoints:* this selects all breakpoints between consecutive DNA copy number segments, except for breakpoints flanked by two copy number neutral segments; C) *CNA-breakpoints*: this selects only those breakpoints flanked by segments with dissimilar discrete DNA copy number states.

Breakpoints are defined by the genomic start position of the copy number segments. DNA copy number profiling data is typically granular due to the distance between microarray probes or bin size of WGS copy number data. This means that the genomic location of a breakpoint is not detected at nucleotide resolution but represents a chromosomal interval with a size that is determined by microarray probe density or WGS bin size.

### Breakpoint gene identification

For identification of genes affected by chromosomal breakpoints the built-in gene annotations can be used. Alternatively, a user-defined gene annotation file can be provided (see Bioconductor vignette and manual for further details). The implemented mapping approach identifies genes that are associated with one or multiple chromosomal breakpoint intervals.

### Cohort-based breakpoint statistics: breakpoint and gene level

Identification of statistically recurrent breakpoint events across all samples can be performed on both chromosomal location- and gene-level. As features, *i.e.* microarray probes or bins of WGS copy number data, are (nearly) equally distributed over the genome, we assume that the null- probability for breakpoint occurrence is equal for all individual candidate breakpoints (features). It differs per sample, though, and equals p _s_ = N _s_/N, where N is the number of probes, and N _s_ the total number of breakpoint for samples. The test statistic is T _p_ is the total number of breakpoints for probe p across all samples. Then, under the null-hypothesis, T _p_ is simply a sum of independent Bernoulli (p _s_) random variables, the null-distribution of which is the same for all probes. It is quickly computed by using probability generating functions, giving also the p-values for any observed value of T _p_.

The probe-based statistical analysis uses Benjamini-Hochberg false discovery rate (FDR) correction for multiple testing. For the intended use at gene level, a more advanced statistical null-model is required. For the gene level, the null-probability for a breakpoint to occur within an individual gene, depends on 1) the length of the gene, 2) the number of gene-associated features and 3) the number of breakpoints in the entire tumor profile for the specific sample. Therefore, at gene-level, we apply a linear regression-based correction for covariates. These regression-estimates are then used as gene- and sample-specific breakpoint null-probabilities (p _g,s_). The test statistic remains the same, and so does the null-distribution computation, although it has to be repeated for each gene now. Finally, the Gilbert FDR correction that accounts for discreteness in the null-distribution ^16^ is applied in this analysis to determine significance of recurrent breakpoint genes. Commands and example workflow can be found in Bioconductor vignette and manual.

## Use case

### Identification of recurrent breakpoint genes in advanced colorectal cancers

We applied our method to 352 high-resolution array-CGH samples from a series of advanced colorectal cancers ^17^ following CNA detection using ‘CGHcall’ ^13^. Array-CGH data are available in the Gene Expression Omnibus database under accession number GSE63216 ( www.ncbi.nlm.nih.gov/projects/geo/). We selected for the CNA-associated breakpoints (setting: ‘CNA-associated’), used gene annotations from ensembl (human genome NCBI build36/hg18, release 54) and applied the dedicated Benjamini-Hochberg-type FDR correction (setting: ‘Gilbert’), for recurrent breakpoint gene identification. A total of 748 genes appeared to be recurrently affected by chromosomal breaks (FDR<0.1) ^5^. Breakpoint frequencies of chromosome 20p are visualized with the built-in plot function ( Figure 1; see Bioconductor vignette and manual for further details about this function). Interestingly, patient stratification based on recurrent gene breakpoints and well-known point mutations by propagation to the predefined STRING human protein interaction network revealed one CRC subtype with very poor prognosis, which supported clinical relevance of this class of somatic aberrations in advanced colorectal cancers ^5^.

## Conclusion

Genome instability including numerical and structural somatic chromosomal aberrations is a hallmark of cancer. Several tools are available that focus on detection of numerical aberrations of large chromosome segments. The R-package ‘GeneBreak’ extracts additional information from CNA data. ‘GeneBreak’ provides an easy-to-use algorithm, which handles identification of genomic breakpoint locations, mapping of breakpoints to genes and includes a comprehensive statistical approach to reveal recurrent breakpoint genes from series of tumor samples. Therefore, ‘GeneBreak’ can be applied to detect CNA-associated chromosomal breaks in individual tumor samples and facilitates detection of recurrent breakpoint genes across multiple tumor samples.

## Data and software availability

Publicly available copy number data used for the use case is deposited at Gene Expression Omnibus database under accession number GSE63216 ( https://protect-eu.mimecast.com/s/6LQhBmNGvCG).

Software available from: *C www.bioconductor.org/packages/release/bioc/html/GeneBreak.html* and https://protect-eu.mimecast.com/s/aLGhBqmpgF2

Latest source code: https://github.com/F1000Research/GeneBreak/releases/tag/v1.0

Archived source code as at the time of publication: F1000Research/Genebreak, doi: 10.5281/zenodo.153937 ^18^

License: GPL 2

## Supplementary material

**GeneBreak vignette.**

.

**GeneBreak Manual.**

## References

[1] Stratton MR Campbell PJ Futreal PA: The cancer genome. *Nature.* 2009;458(7239):719–724. 10.1038/nature07943 19360079 PMC2821689

[2] Forbes SA Bindal N Bamford S: COSMIC: mining complete cancer genomes in the Catalogue of Somatic Mutations in Cancer. *Nucleic Acids Res.* 2011;39(Database issue):D945–D950. 10.1093/nar/gkq929 20952405 PMC3013785

[3] Mitelman F Johansson B Mertens F: The impact of translocations and gene fusions on cancer causation. *Nat Rev Cancer.* 2007;7(4):233–245. 10.1038/nrc2091 17361217

[4] Inaki K Liu ET: Structural mutations in cancer: mechanistic and functional insights. *Trends Genet.* 2012;28(11):550–559. 10.1016/j.tig.2012.07.002 22901976

[5] van den Broek E Dijkstra MJ Krijgsman O: High Prevalence and Clinical Relevance of Genes Affected by Chromosomal Breaks in Colorectal Cancer. *PLoS One.* 2015;10(9):e0138141. 10.1371/journal.pone.0138141 26375816 PMC4574474

[6] Malhotra A Lindberg M Faust GG: Breakpoint profiling of 64 cancer genomes reveals numerous complex rearrangements spawned by homology-independent mechanisms. *Genome Res.* 2013;23(5):762–776. 10.1101/gr.143677.112 23410887 PMC3638133

[7] Edwards PA: Fusion genes and chromosome translocations in the common epithelial cancers. *J Pathol.* 2010;220(2):244–254. 10.1002/path.2632 19921709

[8] Hermsen M Snijders A Guervós MA: Centromeric chromosomal translocations show tissue-specific differences between squamous cell carcinomas and adenocarcinomas. *Oncogene.* 2005;24(9):1571–1579. 10.1038/sj.onc.1208294 15674345

[9] Muggeo VM Adelfio G: Efficient change point detection for genomic sequences of continuous measurements. *Bioinformatics.* 2011;27(2):161–166. 10.1093/bioinformatics/btq647 21088029

[10] Ritz A Paris PL Ittmann MM: Detection of recurrent rearrangement breakpoints from copy number data. *BMC Bioinformatics.* 2011;12:114. 10.1186/1471-2105-12-114 21510904 PMC3112242

[11] Toloşi L Theißen J Halachev K: A method for finding consensus breakpoints in the cancer genome from copy number data. *Bioinformatics.* 2013;29(14):1793–1800. 10.1093/bioinformatics/btt300 23716195

[12] Liu H Zilberstein A Pannier P: Evaluating translocation gene fusions by SNP array data. *Cancer Inform.* 2012;11:15–27. 10.4137/CIN.S8026 22259228 PMC3256939

[13] van de Wiel MA Kim KI Vosse SJ: CGHcall: calling aberrations for array CGH tumor profiles. *Bioinformatics.* 2007;23(7):892–894. 10.1093/bioinformatics/btm030 17267432

[14] Scheinin I Sie D Bengtsson H: DNA copy number analysis of fresh and formalin-fixed specimens by shallow whole-genome sequencing with identification and exclusion of problematic regions in the genome assembly. *Genome Res.* 2014;24(12):2022–2032. 10.1101/gr.175141.114 25236618 PMC4248318

[15] Olshen AB Venkatraman ES Lucito R: Circular binary segmentation for the analysis of array-based DNA copy number data. *Biostatistics.* 2004;5(4):557–572. 10.1093/biostatistics/kxh008 15475419

[16] Gilbert PB: A modified false discovery rate multiple-comparisons procedure for discrete data, applied to human immunodeficiency virus genetics. *Appl Statist.* 2005;54(1):143–158. 10.1111/j.1467-9876.2005.00475.x

[17] Haan JC Labots M Rausch C: Genomic landscape of metastatic colorectal cancer. *Nat Commun.* 2014;5: 5457. 10.1038/ncomms6457 25394515 PMC4243240

[18] Broek E: F1000Research/GeneBreak. *Zenodo*. 2016 Data Source

