# Supplementary material for: GeneBreak: detection of recurrent DNA copy number aberration-associated chromosomal breakpoints within genes
Source: F1000Res. 2017 Jul 6;5:2340. Originally published 2016 Sep 19. [Version 2] doi: 10.12688/f1000research.9259.2 (PMC5500957; doi:10.12688/f1000research.9259.2)
Supplement: Supplementary file 1 [file f1000research-5-12600-s0000.tgz › b9923769-65e7-4664-819e-73db3794f9b5.pdf]

Introduction to GeneBreak

Evert van den Broek*
& Stef van Lieshout

October 14, 2015

Department of Pathology
VU University Medical Center
The Netherlands, Amsterdam

Contents

1 Running GeneBreak **2**
1.1 Detect breakpoints from copy-number data 2
1.1.1 Loading cghCall object 2
1.1.2 Loading data from a dataframe 3
1.2 Breakpoint selection by filtering 4
1.3 Identification of genes affected by breakpoints 4
1.3.1 Loading gene annotation data 4
1.3.2 Feature-to-gene mapping 5
1.3.3 Detection of gene-associated breakpoints 6
1.4 Cohort-based breakpoint statistics 6
1.4.1 Detection of recurrent breakpoint genes 7
1.4.2 Detection of recurrent breakpoint locations 7
1.5 Visualization of breakpoint frequencies 8

2 Storage of R objects **10**

3 Downloading Gene Annotations **10**

4 Session Information **12**

*Correspondence to: Christian Rausch (c.rausch@vumc.nl) or Sanne Abeln (s.abeln@vu.nl)

1 Running GeneBreak

The `GeneBreak` package aims to systematically identify genes recurrently affected by copy number aberration-associated breakpoint locations that indicate underlying DNA breaks and thereby genes involved in structural variants. This is a short tutorial on how to use the `GeneBreak` package. It describes an example workflow which uses copy number aberration (CNA) data obtained by analysis of 200 array-CGH (Agilent 180k) samples from advanced colorectal cancers. We used the `CGHcall` package that can be obtained via Bioconductor (www.bioconductor.org). First, we will start with loading the package.

```
> library(GeneBreak)
```

1.1 Detect breakpoints from copy-number data

Copy number data can be loaded in two ways. We recommend the usage of Bioconductor packages `CGHcall` or `QDNAseq` to process CNA data from array-CGH or sequencing data respectively. The obtained cghCall/QDNAseq object can directly serve as input for the GeneBreak pipeline. Alternatively, a data.frame with exactly these five columns: "Chromosome", "Start", "End" and "FeatureName" (usually probe or bin identifier) followed by columns with sample data can be provided. In this tutorial we will use a built-in dataset that contains CNA data from chromosome 20:

1.1.1 Loading cghCall object

To load and run the example dataset, which is an object of class `CGHcall`, the `CGHcall` package needs to be installed.

```
> # Install the "CGHcall" package from Bioconductor:
> # source("https://bioconductor.org/biocLite.R")
> # biocLite("CGHcall")
```

Load the example dataset from `GeneBreak`:

```
> library(CGHcall)
> data( "copynumber.data.chr20" )
```

Inspection of the loaded data shows an R object of class `cghCall` that contains CNA data from 3653 features (array-CGH probes in this case) and 200 samples.

```
> copynumber.data.chr20

cghCall (storageMode: lockedEnvironment)
assayData: 3653 features, 200 samples
  element names: calls, copynumber, probamp, probgain, probloss, probnorm, segmented
protocolData: none
```

```
phenoData
  sampleNames: sample_1 sample_2 ... sample_200 (200 total)
  varLabels: Cellularity
  varMetadata: labelDescription
featureData
  featureNames: A_16_P03469195 A_14_P136138 ... A_18_P13856091
    (3653 total)
  fvarLabels: Chromosome Start End
  fvarMetadata: labelDescription
experimentData: use 'experimentData(object)'
Annotation:
```

To generate an object of class `CopyNumberBreakpoints` with breakpoint locations, run `getBreakpoints()`. This will obtain the required information from the `cghCall` object and determine the breakpoint locations.

```
> breakpoints <- getBreakpoints( data = copynumber.data.chr20 )
```

```
Breakpoint detection started...
```

```
> breakpoints
```

```
 --- Object Info ---
 This is an object of class "CopyNumberBreakPoints"
 3653 features by 200 samples
 A total of 1035 breakpoints
 See accessOptions(object) for how to access data in this object
```

Inspection of the generated object shows that we have copy number data of 3653 features from 200 samples. A total of 1035 individual breakpoint locations were identified.

1.1.2 Loading data from a dataframe

If the CNA data has not been generated by `CGHcall` or `QDNAseq`, there is a possibilty of using a `data.frame()` as input for `GeneBreak`. This allows breakpoint analysis of data from any copy number detection pipeline by importing a text file into `getBreakpoints()`.

Here we show how to use two data.frames() with segment and (optionally) call values as input for `getBreakpoints` instead of a cghCall/QDNAseq object.

```
> library(CGHcall)
> cgh <- copynumber.data.chr20
> segmented <- data.frame( Chromosome=chromosomes(cgh), Start=bpstart(cgh),
+  End=bpend(cgh), FeatureName=rownames(cgh), segmented(cgh))
> called <- data.frame( Chromosome=chromosomes(cgh), Start=bpstart(cgh),
+  End=bpend(cgh), FeatureName=rownames(cgh), calls(cgh))
> breakpoints <- getBreakpoints( data = segmented, data2 = called )
```

Note: the first five column names of the data.frame must exactly be ”Chromosome”, ”Start”, ”End” and ”FeatureName”.

1.2 Breakpoint selection by filtering

Next, breakpoints can be filtered by stringent criteria. Different filters can be set (see `?bpFilter` for more details). Default setting is ”CNA-ass” which means that breakpoints flanked by copy number neutral segments will be filtered out. Note: you need discrete copy number calls (loss,neutral, gain, etc) for this option.

```
> breakpointsFiltered <- bpFilter( breakpoints, filter = "CNA-ass" )

Applying BP selection...

> breakpointsFiltered

 --- Object Info ---
 This is an object of class "CopyNumberBreakPoints"
 3653 features by 200 samples
 A total of 985 breakpoints
 See accessOptions(object) for how to access data in this object
```

Inspection of the output shows that 985 CNA-associated breakpoint locations remain following the filter step.

1.3 Identification of genes affected by breakpoints

Identification of genes affected by breakpoints requires execution of the following three steps.

1.3.1 Loading gene annotation data

We need to load gene annotations to be able to identify genes affected by breakpoints in the next step. Gene annotation for human reference genome hg18 (and hg19, hg38) are built-in, but also user-defined annotations can be used. The required columns for this data.frame are ”Gene”, ”Chromosome”, ”Start” and ”End”.

```
> data( "ens.gene.ann.hg18" )
```

This shows the content of the first six rows of the hg18 gene annotation dataframe:

```
> head( ens.gene.ann.hg18 )

             Gene           EnsID Chromosome  Start    End   band strand
21297 MIRN1302-2 ENSG00000221311          1  20229  20366 p36.33      1
21       FAM138E ENSG00000222027          1  24417  25944 p36.33     -1
827      FAM138E ENSG00000222003          1  24417  25944 p36.33     -1
828      FAM138A ENSG00000222003          1  24417  25944 p36.33     -1
829        OR4F5 ENSG00000177693          1  58954  59871 p36.33      1
830       OR4F29 ENSG00000177799          1 357522 358460 p36.33      1
```

1.3.2 Feature-to-gene mapping

Here, the loaded gene annotation information will be added to the GeneBreak object and feature-to-gene mapping will be performed.

```
> breakpointsAnnotated <- addGeneAnnotation( breakpointsFiltered, ens.gene.ann.hg18 )
```

```
Adding of gene annotation started on 659 genes by 200 samples
0% ... 25% ... 50% ... 75% ... Adding gene annotation DONE
```

Tho show the names of associated features of e.g. the "PCMTD2" gene, give:

```
> featuresPerGene ( breakpointsAnnotated , geneName = "PCMTD2" )
```

```
Gene chosen: PCMTD2
[1] "A_14_P125849"   "A_16_P21189265" "A_16_P21189294" "A_16_P34766035"
```

Gene-associated feature information has been added to `breakpointsAnnotated`. Visualisation shows:

```
> geneFeatures <- geneInfo( breakpointsAnnotated )
> head( geneFeatures[ ,
+   c("Gene", "Chromosome", "Start", "End", "featureTotal",
+     "featureNames", "remarks") ] )
```

```
        Gene Chromosome  Start    End featureTotal
1366 DEFB125         20  16351  25296            2
1376 DEFB126         20  71231  74391            2
1383 DEFB127         20  86122  87804            2
1393 DEFB128         20 116527 118264            1
1396 DEFB129         20 155899 158523            2
1402 DEFB132         20 186377 189735            2
                      featureNames remarks
1366 A_14_P136138,A_16_P03469215       D
1376   A_14_P122034,A_14_P106962       D
1383 A_14_P106962,A_16_P41238845       D
1393              A_16_P41238870       C
1396 A_14_P113156,A_16_P03469327       D
1402 A_14_P200562,A_16_P41239011       D
```

Possible "remarks" that describe gene position with respect to feature positions are: "A": genes located upstream of the first chromosomal feature (no gene-associated features) "B": genes located downstream of the last chromosomal feature (no gene-associated features) "C": in case of array-CGH probes, the whole gene is located between two features "C": in case of sequencing data, the whole gene is located between start and end of one bin "D": gene represented by one or multiple features "E": gene represented by one or multiple features, but the end of the gene is not covered by any feature "X": no feature covers the chromosome of the gene

1.3.3 Detection of gene-associated breakpoints

In the next step, gene-associated breakpoints will be identified by using `bpGenes()`.

```
> breakpointGenes <- bpGenes( breakpointsAnnotated )
```

```
Running bpGenes: 659 genes and 200 samples
0% ... 25% ... 50% ... 75% ... bpGenes DONE
A total of 1029 gene breaks in 241 genes detected
```

This is an example of the output when selected for broken genes:

```
> result_BreakpointGenes <- geneInfo ( breakpointGenes )
> head( result_BreakpointGenes[ which ( result_BreakpointGenes$sampleCount > 0 ) ,
+   c( "Gene", "Chromosome", "Start", "End", "featureTotal", "nrOfBreakLocations",
+      "sampleCount", "sampleNamesWithBreakpoints") ] )
```

```
         Gene Chromosome   Start     End featureTotal nrOfBreakLocations
1414 C20orf96         20  199504  219390            3                  1
1633    SRXN1         20  575270  581890            2                  1
1643    SCRT2         20  590241  604823            2                  1
1683    RSPO4         20  887098  930904            4                  1
1694    PSMF1         20 1041906 1097022            5                  3
1808   NSFL1C         20 1370807 1396417            3                  1
     sampleCount                              sampleNamesWithBreakpoints
1414           1                                               sample_60
1633           1                                              sample_128
1643           1                                              sample_128
1683           1                                                sample_1
1694           5 sample_16,sample_42,sample_105,sample_180,sample_195
1808           1                                               sample_64
```

This table shows the genes (rows) and the number of gene-associated features in ”featureTotal”. The column ”nrOfBreakLocations” indicates the number of identified breakpoint locations in the gene across all samples. As a consequence, this is a subset of, and limited by, the total number of gene-associated features. The total of samples that harbor a breakpoint in the gene is given in the column ”sampleCount”.

1.4 Cohort-based breakpoint statistics

Following identification of (gene) breakpoints per profile, breakpoint events that are significantly recurring across samples will be determined by dedicated statistical analysis. This can be performed at ”gene” (breakpoint gene) and/or ”feature” (breakpoint location) level. Two different methods of FDR-type correction for multiple testing can be used, the standard Benjamini-Hochberg FDR-type correction (”BH”) or dedicated Benjamini-Hochberg FDR-type correction (”Gilbert”).

1.4.1 Detection of recurrent breakpoint genes

The gene-based statistical analysis includes correction for covariates that may influence the probability to be a breakpoint gene including number of breakpoints in a profile, number of gene-associated features and gene length by gene-associated feature coverage. Multiple testing can be applied by the powerful dedicated Benjamini-Hochberg FDR-type correction ("Gilbert") that accounts for the discreteness of the null-distribution. (Reference: Gilbert PB, Appl Statist. 2005;54:143-58) NOTE: when running `bpStats()` warnings can be generated by a function (glm.fit) of a dependancy package, this does not harm the analysis.

```
> breakpointStatistics <- bpStats( breakpointGenes,
+   level = "gene", method = "Gilbert" )
```

```
Applying statistical test over 200 samples for: gene breakpoints: Gilbert test...
```

This will return an object of class `CopyNumberBreakPointGenes`. By using `recurrentGenes()` we can observe the recurrent affected genes with associated P-value and FDR.

```
> head( recurrentGenes( breakpointStatistics ) )
```

```
 A total of 19 recurrent breakpoint genes (at FDR < 0.1)
          Gene sampleCount featureTotal        pvalue          FDR
13886   PCMTD2          64            4 1.350385e-103 1.848343e-101
13898 C20orf69          33            3  5.522293e-44  3.860197e-42
4268     BFSP1           8            5  3.941447e-07  3.148759e-05
5473    ABHD12          10            9  5.756361e-05  3.687639e-03
4780  C20orf26           7           18  2.748743e-04  1.204846e-02
4102    KIF16B           7           19  4.054266e-04  1.322722e-02
```

1.4.2 Detection of recurrent breakpoint locations

With this step, statistics at breakpoint location (feature) level will be added to the object of class `CopyNumberBreakPointGenes`. Here, we recommend to use the less computationally intensive standard Benjamini-Hochberg FDR-type correction for multiple testing, because the breakpoint probability is equal across features per profile, which means that all positions correspond to the same null-distribution.

```
> breakpointStatistics <- bpStats(
+   breakpointStatistics, level = "feature", method = "BH" )
```

```
Applying statistical test over 200 samples for feature breakpoints: BH test...
```

```
> breakpointStatistics
```

```
--- Object Info ---
This is an object of class "CopyNumberBreakPointGenes"
3653 features by 200 samples
A total of 985 breakpoints
A total of 1029 gene breaks in 241 genes
A total of 19 recurrent breakpoint genes (FDR < 0.1)
A total of 29 recurrent breakpoints (FDR < 0.1)
See accessOptions(object) for how to access data in this object
```

By using `featureInfo()` we can observe the features and whether they were identified as breakpoints including the calculated FDR values:

```
> head( featureInfo( breakpointStatistics ) )

               Chromosome Start   End featureInterval sampleCount
A_16_P03469195         20  8747  8793               0           0
A_14_P136138           20 18580 18639            9833           0
A_16_P03469215         20 25530 25589            6950           0
A_16_P21047338         20 32699 32743            7169           0
A_16_P41238750         20 39125 39184            6426           0
A_16_P03469235         20 50422 50481           11297           0
               sampleNamesWithBreakpoints nrOfBreakLocations pvalue FDR
A_16_P03469195                                             0      1   1
A_14_P136138                                               0      1   1
A_16_P03469215                                             0      1   1
A_16_P21047338                                             0      1   1
A_16_P41238750                                             0      1   1
A_16_P03469235                                             0      1   1
```

1.5 Visualization of breakpoint frequencies

Breakpoint locations and frequencies can be visualized using `bpPlot()`:

```
> bpPlot( breakpointStatistics, fdr.threshold = 0.1 )

Plotting breakpoint frequencies ...
Plotting Chromosome: 20
```

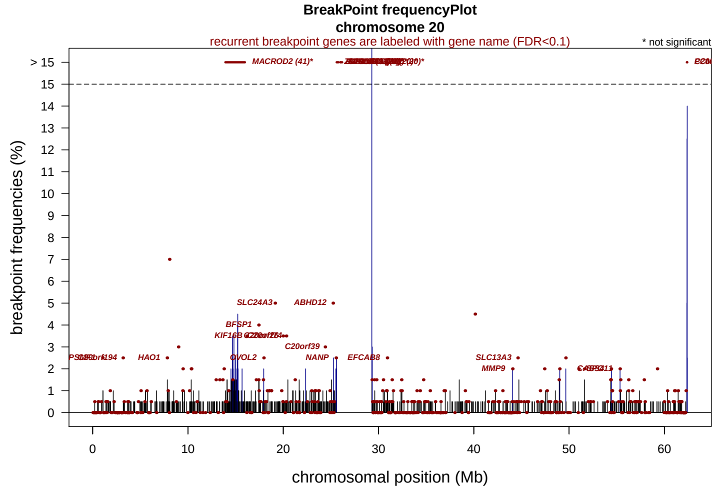

Figure 1: Graphical representation of CNA-associated chromosomal breakpoint frequencies and their distribution over chromosomes 20. The X-axis depicts the genomic position in Mb. The Y-axis depicts the chromosomal breakpoint frequencies across the series of 200 CRC samples. Breakpoint frequencies are indicated on array-CGH probe-level (vertical black bars) and on gene-level (horizontal red bars). Recurrent breakpoint genes (FDR<0.1) are named. When the gene breakpoint frequency exceeded 15% (horizontal dashed line), the breakpoint frequency (%) follows the gene name.

2 Storage of R objects

At any time during the analysis, the GeneBreak objects (and any R objects for that matter) can be saved to disk with: `saveRDS`, and in the future be read from the local file with `loadRDS`

3 Downloading Gene Annotations

This section describes the steps taken to create the gene annotations used in this package. It may serve as a start for creating your own if required for whatever reason.

```
> # gene annotations obtained via Biomart.
> # HUGO gene names (HGNC symbol), Ensembl_ID and chromosomal location
>
> # Used (and most) recent releases:
> # HG18: release54
> # HG19: release75
> # HG38: release80 (date: 150629)
>
> library(biomaRt)
> ensembl54 = useMart(
+   host = 'may2009.archive.ensembl.org',
+   biomart = 'ENSEMBL_MART_ENSEMBL',
+   dataset = "hsapiens_gene_ensembl"
+ )
> ensembl75 = useMart(
+   host = 'feb2014.archive.ensembl.org',
+   biomart = 'ENSEMBL_MART_ENSEMBL',
+   dataset = "hsapiens_gene_ensembl"
+ )
> ensembl80 = useMart(
+   "ensembl",
+   dataset = "hsapiens_gene_ensembl"
+ )
> createAnnotationFile <- function( biomartVersion ) {
+   biomart_result <- getBM(
+     attributes =  c(
+       "hgnc_symbol", "ensembl_gene_id", "chromosome_name",
+       "start_position", "end_position", "band", "strand"
+     ),
+     mart = biomartVersion
+   )
+
+   biomart_result[ ,3] <- as.vector( biomart_result[ ,3] )
+   idx_x <- biomart_result$chromosome_name == "X"
```

```
+   idx_y <- biomart_result$chromosome_name == "Y"
+   biomart_result$chromosome_name[ idx_x ] <- "23"
+   biomart_result$chromosome_name[ idx_y ] <- "24"
+
+   biomart_genes <- biomart_result[ which(biomart_result[ ,1] != "" &
+     biomart_result[ ,3] %in% c(1:24)) , ]
+   colnames(biomart_genes)[1:5] <- c("Gene","EnsID","Chromosome","Start","End")
+
+   cat(
+     c( "Biomart version:", biomartVersion@host,
+           "including:", dim(biomart_genes)[1], "genes\n"
+     )
+   )
+
+   return( biomart_genes )
+ }
> ens.gene.ann.hg18 <- createAnnotationFile( ensembl54 )
> ens.gene.ann.hg19 <- createAnnotationFile( ensembl75 )
> ens.gene.ann.hg38 <- createAnnotationFile( ensembl80 )
>
```

4 Session Information

The version number of R and packages loaded for generating the vignette were:

```
R version 3.2.2 (2015-08-14)
Platform: x86_64-pc-linux-gnu (64-bit)
Running under: Ubuntu 14.04.3 LTS

locale:
 [1] LC_CTYPE=en_US.UTF-8       LC_NUMERIC=C
 [3] LC_TIME=en_US.UTF-8        LC_COLLATE=C
 [5] LC_MONETARY=en_US.UTF-8    LC_MESSAGES=en_US.UTF-8
 [7] LC_PAPER=en_US.UTF-8       LC_NAME=C
 [9] LC_ADDRESS=C               LC_TELEPHONE=C
[11] LC_MEASUREMENT=en_US.UTF-8 LC_IDENTIFICATION=C

attached base packages:
[1] stats4    parallel  stats     graphics  grDevices utils     datasets
[8] methods   base

other attached packages:
 [1] GeneBreak_1.0.0      GenomicRanges_1.22.0 GenomeInfoDb_1.6.0
 [4] IRanges_2.4.0        S4Vectors_0.8.0      CGHcall_2.32.0
 [7] snowfall_1.84-6      snow_0.3-13          CGHbase_1.30.0
[10] marray_1.48.0        limma_3.26.0         Biobase_2.30.0
[13] BiocGenerics_0.16.0  DNAcopy_1.44.0       impute_1.44.0
[16] QDNAseq_1.6.0

loaded via a namespace (and not attached):
 [1] XVector_0.10.0       zlibbioc_1.16.0      BiocParallel_1.4.0
 [4] tools_3.2.2          R.oo_1.19.0          lambda.r_1.1.7
 [7] futile.logger_1.4.1  matrixStats_0.14.2   R.utils_2.1.0
[10] futile.options_1.0.0 bitops_1.0-6         R.methodsS3_1.7.0
[13] Rsamtools_1.22.0     Biostrings_2.38.0
```
