# Supplementary material for: GeneBreak: detection of recurrent DNA copy number aberration-associated chromosomal breakpoints within genes
Source: F1000Res. 2017 Jul 6;5:2340. Originally published 2016 Sep 19. [Version 2] doi: 10.12688/f1000research.9259.2 (PMC5500957; doi:10.12688/f1000research.9259.2)
Supplement: Supplementary file 2 [file f1000research-5-12600-s0001.tgz › b0fbf3c2-13c5-4803-9f0b-ae37f1e3e412.pdf]

Package ‘GeneBreak’

October 27, 2015

Type Package

Version 1.0.0

Title Gene Break Detection

Author Evert van den Broek, Stef van Lieshout

Maintainer Evert van den Broek <vandenbroek.evert@gmail.com>

Imports graphics, methods

Depends R(>= 3.2), QDNAseq, CGHcall, CGHbase, GenomicRanges

URL https://github.com/stefvanlieshout/GeneBreak

Description Recurrent breakpoint gene detection on copy number aberration profiles.

License GPL-2

biocViews aCGH, CopyNumberVariation, DNASeq, Genetics, Sequencing, WholeGenome, Visualization

LazyData FALSE

NeedsCompilation no

R topics documented:

accessOptions,CopyNumberBreakPoints-method 2
addGeneAnnotation,CopyNumberBreakPoints-method 3
bpFilter,CopyNumberBreakPoints-method 4
bpGenes,CopyNumberBreakPointGenes-method 5
bpPlot,CopyNumberBreakPoints-method 5
bpStats,CopyNumberBreakPoints-method 6
breakpointData,CopyNumberBreakPoints-method 8
breakpointsPerGene,CopyNumberBreakPointGenes-method 8
callData,CopyNumberBreakPoints-method 9
copynumber.data.chr18 9
copynumber.data.chr20 10
copynumber.data.chr21 10
CopyNumberBreakPointGenes-class 11
CopyNumberBreakPoints-class 12
ens.gene.ann.hg18 13
ens.gene.ann.hg19 14
ens.gene.ann.hg38 14

featureChromosomes,CopyNumberBreakPoints-method 15
featureInfo,CopyNumberBreakPoints-method 16
featuresPerGene,CopyNumberBreakPointGenes-method 16
GeneBreak 17
geneChromosomes,CopyNumberBreakPointGenes-method 17
geneInfo,CopyNumberBreakPointGenes-method 18
getBreakpoints 19
namesFeatures,CopyNumberBreakPoints-method 20
recurrentGenes,CopyNumberBreakPointGenes-method 21
sampleNames,CopyNumberBreakPoints-method 21
segmentData,CopyNumberBreakPoints-method 22

Index **23**

`accessOptions,CopyNumberBreakPoints-method`
Access Object Data. This method lists possible functions to access the data of the object.

Description

Access Object Data. This method lists possible functions to access the data of the object.

Usage

```
## S4 method for signature 'CopyNumberBreakPoints'
accessOptions(object)
```

Arguments

`object`	An object of class `CopyNumberBreakPoints` or `CopyNumberBreakPointGenes`

Value

prints text to screen

Examples

```
data( copynumber.data.chr20 )
bp <- getBreakpoints( copynumber.data.chr20 )

accessOptions( bp )
```

addGeneAnnotation,CopyNumberBreakPoints-method
addGeneAnnotation

Description

Maps features to gene locations.

Usage

```
## S4 method for signature 'CopyNumberBreakPoints'
addGeneAnnotation(object, geneAnnotation)
```

Arguments

`object`	An object of class CopyNumberBreakPoints
`geneAnnotation`	An object of class `GRanges` or dataframe with at least four columns ("Gene", "Chromosome", "Start", "End")

Details

The end of the first feature after gene start location up to and including the first feature after gene end location will be defined as gene-associated feaures. For hg18, hg19 and hg38 built-in gene annotation files obtained from ensembl can be used. Please take care to use a matching reference genome for your breakpoint data. In stead of using the built-in gene annotion files, feature-to-gene mapping can be based on an user-defined annotion file. The dataframe should contain at least these four columns: "Gene", "Chromosome", "Start" and "End".

Value

Returns an object of class CopyNumberBreakPointGenes with gene annotation added.

Examples

```
data( copynumber.data.chr20 )
data( ens.gene.ann.hg18 )

## other buil-in gene annotations are:
# data( ens.gene.ann.hg19 )
# data( ens.gene.ann.hg38 )

bp <- getBreakpoints( copynumber.data.chr20 )
bp <- bpFilter( bp )
# input copynumber.data.chr20 is hg18 based
bp <- addGeneAnnotation( bp, ens.gene.ann.hg18 )

## options to inspect the data
bp
accessOptions( bp )
```

`bpFilter,CopyNumberBreakPoints-method`
bpFilter

Description

Selects breakpoints by filter criteria options.

Usage

```
## S4 method for signature 'CopyNumberBreakPoints'
bpFilter(object, filter = "CNA-ass",
  threshold = NULL)
```

Arguments

`object`	An object of class CopyNumberBreakPoints
`filter`	Type of filter. This can be either "CNA-ass", "deltaSeg" or "deltaCall".<br>• CNA-ass: filter out breakpoints that are flanked by copy number neutral segments to obtain CNA-associated breakpoint locations<br>• deltaSeg: selects for breakpoints where the log2 ratio transition of the copy number segments exceeds the user-defined threshold<br>• deltaCall: selects only breakpoints of discrete copy number states (amplification, gain, neutral, loss)
`threshold`	Set the minimal log2 ratio difference between segments. This parameter is required for the "deltaSeg" filter option

Details

Filter options "CNA-ass" and "deltaCall" require calls in addition to segmented copynumber data (see input for `getBreakpoints()`)

Value

Returns an object of class CopyNumberBreakPoints with breakpoint matrix replaced by filtered breakpoints.

Examples

```
data( copynumber.data.chr20 )
bp <- getBreakpoints( copynumber.data.chr20 )
bp <- bpFilter( bp, filter = "CNA-ass" )
bp <- bpFilter( bp, filter = "deltaSeg", threshold = 0.2 )

## options to inspect the data
bp
accessOptions( bp )
```

bpGenes,CopyNumberBreakPointGenes-method

bpGenes

Description

Indentifies genes affected by breakpoint locations.

Usage

```
## S4 method for signature 'CopyNumberBreakPointGenes'
bpGenes(object)
```

Arguments

object An object of class CopyNumberBreakPointGenes

Details

This step requires feature-to-gene annotations added to the input object (see `?addGeneAnnotation`).

Value

Returns an object of class CopyNumberBreakPointGenes with gene-breakpoint information

Examples

```
data( copynumber.data.chr20 )
data( ens.gene.ann.hg18 )

bp <- getBreakpoints( copynumber.data.chr20 )
bp <- bpFilter( bp )
bp <- addGeneAnnotation( bp, ens.gene.ann.hg18 )
bp <- bpGenes( bp )

## options to inspect the data
bp
accessOptions( bp )
```

bpPlot,CopyNumberBreakPoints-method

bpPlot

Description

Plots breakpoint frequencies per chromosome

Usage

```
## S4 method for signature 'CopyNumberBreakPoints'
bpPlot(object, plot.chr = NULL,
  plot.ylim = 15, fdr.threshold = 0.1, add.jitter = FALSE)
```

Arguments

object	An object of class CopyNumberBreakPoints or CopyNumberBreakPointGenes
plot.chr	A vector with chromosome(s) to plot. All chromosomes will be plotted when NULL is used.
plot.ylim	An integer giving the max y coordinate.
fdr.threshold	The FDR threshold to label recurrent breakpoint genes with their gene name
add.jitter	Logical. If TRUE, function jitter will be used for the y position of gene labels

Details

The plot includes breakpoint locations and breakpoint gene frequencies. Genes that are recurrently affected are labeled with their gene name.

Value

calls plot function

Examples

```
data( copynumber.data.chr20 )
data( ens.gene.ann.hg18 )
bp <- getBreakpoints( copynumber.data.chr20 )
bp <- bpFilter( bp )
bp <- addGeneAnnotation( bp, ens.gene.ann.hg18 )
bp <- bpGenes( bp )
bp <- bpStats( bp )

bpPlot( bp, c(20) )
```

---

bpStats,CopyNumberBreakPoints-method

bpStats

Description

Applies cohort-based statistics to identify genes and/or chromosomal locations that are recurrently affected by breakpoints.

Usage

```
## S4 method for signature 'CopyNumberBreakPoints'
bpStats(object, level = "gene",
  method = "BH", fdr.threshold = 1)
```

Arguments

`object`	An object of class CopyNumberBreakPointGenes
`level`	The level at which to operate, this can be either "gene" (correcting for gene length) or "feature" (per probe/bin)
`method`	The FDR correction method to apply. This can be "BH" (applies Benjamini-Hochberg-type FDR correction) or "Gilbert" (for dedicated Benjamini-Hochberg-type FDR correction)
`fdr.threshold`	The threshold for FDR correction'

Details

The statistical method on gene-level corrects for covariates that may influence the probability to be a breakpoint gene including number of breakpoints in a profile, number of gene-associated features and gene length by gene-associated feature coverage. The statistical analysis includes multiple testing where standard Benjamini-Hochberg-type FDR correction will be performed by default. This less computational intensive method assumes a similar null-distribution for all candidate breakpoint events and satisfies for analysis on breakpoint location-level. For statistics on gene-level however, we recommend to apply the more comprehensive and powerful dedicated Benjamini-Hochberg-type FDR correction that accounts for discreteness in null-distribution (Gilbert, 2005) following correction for covariates that may influence the probability to be a breakpoint gene including number of breakpoints in a profile, number of gene-associated features and gene length by gene-associated feature coverage.

Value

Returns an object of class CopyNumberBreakPointGenes with cohort based statistics added.

References

Gilbert,P.B. (2005) A modified false discovery rate multiple-comparisons procedure for discrete data, applied to human immunodeficiency virus genetics. Journal of the Royal Statistical Society Series C-Applied Statistics, 54, 143-158.

Examples

```
data( copynumber.data.chr20 )
data( ens.gene.ann.hg18 )
bp <- getBreakpoints( copynumber.data.chr20 )
bp <- bpFilter( bp )
bp <- addGeneAnnotation( bp, ens.gene.ann.hg18 )
bp <- bpGenes( bp )
bp <- bpStats( bp )

## options to inspect the data
bp
accessOptions( bp )
```

breakpointData,CopyNumberBreakPoints-method

Access Object breakpointData. This method returns a dataframe with breakpoint values per feature.

Description

Access Object breakpointData. This method returns a dataframe with breakpoint values per feature.

Usage

```
## S4 method for signature 'CopyNumberBreakPoints'
breakpointData(object)
```

Arguments

object	An object of class CopyNumberBreakPoints

Value

a dataframe with breakpoint values

Examples

```
data( copynumber.data.chr20 )
bp <- getBreakpoints( copynumber.data.chr20 )

breakpointData( bp )
```

breakpointsPerGene,CopyNumberBreakPointGenes-method

Access Object breakpointsPerGene. This method returns a dataframe with breakpoints per gene.

Description

Access Object breakpointsPerGene. This method returns a dataframe with breakpoints per gene.

Usage

```
## S4 method for signature 'CopyNumberBreakPointGenes'
breakpointsPerGene(object)
```

Arguments

object	An object of class CopyNumberBreakPoints

Value

a dataframe with breakpoints per gene

Examples

```
data( copynumber.data.chr20 )
data( ens.gene.ann.hg18 )
bp <- getBreakpoints( copynumber.data.chr20 )
bp <- bpFilter( bp )
bp <- addGeneAnnotation( bp, ens.gene.ann.hg18 )
bp <- bpGenes( bp )

breakpointsPerGene( bp )
```

---

callData,CopyNumberBreakPoints-method

Access Object callData. This method returns a dataframe with feature call values.

Description

Access Object callData. This method returns a dataframe with feature call values.

Usage

```
## S4 method for signature 'CopyNumberBreakPoints'
callData(object)
```

Arguments

object	An object of class CopyNumberBreakPoints

Value

a dataframe with feature call values

Examples

```
data( copynumber.data.chr20 )
bp <- getBreakpoints( copynumber.data.chr20 )

callData( bp )
```

---

copynumber.data.chr18

CGHcall Example copynumber data hg18 chr18

Description

A test dataset containing copynumber data of chromosome 18 for the GeneBreak package (hg18 based). This copy number aberration (CNA) data was obtained by analysis of 200 array-CGH (Agilent 180k) samples from advanced colorectal cancers.

Usage

```
data( copynumber.data.chr18 )
```

Format

An object of class cghCall

Value

An object of class cghCall

---

copynumber.data.chr20 *CGHcall Example copynumber data hg18 chr20*

Description

A test dataset containing chromosome 20 copynumber data for the GeneBreak package (hg18 based). This copy number aberration (CNA) data was obtained by analysis of 200 array-CGH (Agilent 180k) samples from advanced colorectal cancers.

Usage

```
data( copynumber.data.chr20 )
```

Format

An object of class cghCall

Value

An object of class cghCall

---

copynumber.data.chr21 *CGHcall Example copynumber data hg18 chr21*

Description

A test dataset containing chromosome 21 copynumber data for the GeneBreak package (hg18 based). This copy number aberration (CNA) data was obtained by analysis of 200 array-CGH (Agilent 180k) samples from advanced colorectal cancers.

Usage

```
data( copynumber.data.chr21 )
```

Format

An object of class cghCall

Value

An object of class cghCall

`CopyNumberBreakPointGenes-class`
An S4 class to represent a CopyNumberBreakPointGenes object

Description

An S4 class to represent a CopyNumberBreakPointGenes object

Slots

`geneAnnotation` A data.frame with original gene annotation input

`geneData` A data.frame with gene information added by package methods

`featuresPerGene` A list with the associated features per gene

`breakpointsPerGene` A matrix with breakage status per gene

Accessors

- `callData( object )` Returns feature call values:
- `segmentData( object )` Returns feature segment values
- `breakpointData( object )` Returns feature breakpoint values
- `sampleNames( object )` Returns vector with sample names
- `namesFeatures( object )` Returns vector with feature names
- `featureChromosomes( object )` Returns vector of feature chromosomes
- `featureInfo( object )` Returns feature data/information
- `geneChromosomes( object )` Returns vector of gene chromosomes
- `geneInfo( object )` Returns gene data/information
- `featuresPerGene( object )` Returns a list of genes with coupled features
- `breakpointsPerGene( object )` Returns gene break status
- `recurrentGenes( object )` Returns recurrently broken genes

Methods

- getBreakpoints Builds the CopyNumberBreakPoints object from copynumber data and detects breakpoint locations
- bpFilter Selects breakpoints by filter criteria options
- addGeneAnnotation Maps features to gene locations
- bpGenes Indentifies genes affected by breakpoint locations
- bpStats Applies cohort-based statistics to identify genes and/or chromosomal locations that are recurrently affected by breakpoints
- bpPlot Plots breakpoint frequencies per chromosome

Author(s)

E. van den Broek and S. van Lieshout

Examples

```
data( copynumber.data.chr20 )
data( ens.gene.ann.hg18 )
bp <- getBreakpoints( copynumber.data.chr20 )
bp <- bpFilter( bp )
bp <- addGeneAnnotation( bp, ens.gene.ann.hg18 )
bp <- bpGenes( bp )
bp <- bpStats( bp )
bpPlot( bp, c(20) )
```

CopyNumberBreakPoints-class
An S4 class to represent a CopyNumberBreakPoints object.

Description

An S4 class to represent a CopyNumberBreakPoints object.

Slots

`segmDiff` A matrix with breakpoints based on segment values

`callDiff` A matrix with breakpoints based on call values

`segments` A matrix with segmented copy number values

`calls` A matrix with copy number calls

`featureAnnotation` A dataframe with predefined information about the features (usually probes or bins)

`featureData` A dataframe with calculated information about the features (usually probes or bins)

Accessors

- callData(object) Returns feature call values
- segmentData(object) Returns feature segment values
- breakpointData(object) Returns feature breakpoint values
- sampleNames(object) Returns vector with sample names
- namesFeatures(object) Returns vector with feature names
- featureChromosomes(object) Returns vector of feature chromosomes
- featureInfo(object) Returns feature data/information

Methods

- getBreakpoints Builds the CopyNumberBreakPoints object from copynumber data and detects breakpoint locations
- bpFilter Selects breakpoints by filter criteria options
- bpStats Applies cohort-based statistics to identify chromosomal locations that are recurrently affected by breakpoints
- bpPlot Plots breakpoint frequencies per chromosome

Author(s)

E. van den Broek and S. van Lieshout

Examples

```
data( copynumber.data.chr20 )
data( ens.gene.ann.hg18 )
bp <- getBreakpoints( copynumber.data.chr20 )
bp <- bpFilter( bp )
bp <- bpStats( bp , level = 'feature' , method = 'BH' )
bpPlot( bp, c(20) )
```

---

ens.gene.ann.hg18 *Gene Annotation hg18*

Description

A dataset containing the gene locations based on human genome reference hg18 that was obtained from BioMart.

Usage

```
data( ens.gene.ann.hg18 )
```

Format

A data.frame

Details

Dataframe with 5 columns:

- Gene: ensembl gene name
- EnsID: ensembl gene id
- Chromosome: Genomic Chromosome
- Start: Genomic start of gene
- End: Genomic end of gene

Value

data.frame

`ens.gene.ann.hg19` *Gene Annotation hg19*

Description

A dataset containing the gene locations based on human genome reference hg19 that was obtained from BioMart.

Usage

```
data( ens.gene.ann.hg19 )
```

Format

A data.frame

Details

Dataframe with 5 columns:

- Gene: ensembl gene name
- EnsID: ensembl gene id
- Chromosome: Genomic Chromosome
- Start: Genomic start of gene
- End: Genomic end of gene

Value

data.frame

`ens.gene.ann.hg38` *Gene Annotation hg38*

Description

A dataset containing the gene locations based on human genome reference hg38 that was obtained from BioMart.

Usage

```
data( ens.gene.ann.hg38 )
```

Format

A data.frame

Details

Dataframe with 5 columns:

- Gene: ensembl gene name
- EnsID: ensembl gene id
- Chromosome: Genomic Chromosome
- Start: Genomic start of gene
- End: Genomic end of gene

Value

data.frame

`featureChromosomes,CopyNumberBreakPoints-method`
Access Object featureChromosomes. This method returns a vector with feature chromosomes.

Description

Access Object featureChromosomes. This method returns a vector with feature chromosomes.

Usage

```
## S4 method for signature 'CopyNumberBreakPoints'
featureChromosomes(object)
```

Arguments

`object`	An object of class `CopyNumberBreakPoints`

Value

a vector with feature chromosomes

Examples

```
data( copynumber.data.chr20 )
bp <- getBreakpoints( copynumber.data.chr20 )
featureChromosomes( bp )
```

`featureInfo,CopyNumberBreakPoints-method`
Access Options featureInfo. This method returns a dataframe with feature annotations.

Description

Access Options featureInfo. This method returns a dataframe with feature annotations.

Usage

```
## S4 method for signature 'CopyNumberBreakPoints'
featureInfo(object)
```

Arguments

object	of class CopyNumberBreakPoints

Value

data.frame with feature annotations

Examples

```
data( copynumber.data.chr20 )
data( ens.gene.ann.hg18 )
bp <- getBreakpoints( copynumber.data.chr20 )
bp <- bpFilter( bp )
bp <- addGeneAnnotation( bp, ens.gene.ann.hg18 )
bp <- bpGenes( bp )

featureInfo( bp )
```

`featuresPerGene,CopyNumberBreakPointGenes-method`
Access Object featuresPerGene. This method returns a vector with gene-related features for a particular gene.

Description

Access Object featuresPerGene. This method returns a vector with gene-related features for a particular gene.

Usage

```
## S4 method for signature 'CopyNumberBreakPointGenes'
featuresPerGene(object, geneName = NULL)
```

Arguments

object	An object of class CopyNumberBreakPoints
geneName	Exact Gene name as in the annotation

Value

a vector with gene-related features

Examples

```
data( copynumber.data.chr20 )
data( ens.gene.ann.hg18 )
bp <- getBreakpoints( copynumber.data.chr20 )
bp <- bpFilter( bp )
bp <- addGeneAnnotation( bp, ens.gene.ann.hg18 )
bp <- bpGenes( bp )

featuresPerGene( bp, geneName="PCMTD2" )
```

GeneBreak *GeneBreak: A package for gene breakpoint detection on copy number abberation data*

Description

The GeneBreak package performs cohort based recurrent gene breakpoint detection on copynumber data. It is possible to use the output of the function CGHcall from the package CGHcall or the function callBins from the package QDNAseq as the input for this analysis.

GeneBreak functions

Analysis starts with the function getBreakpoints and continues with:
bpFilter to exclude certain breakpoints from the analysis
addGeneAnnotation to add gene location information
bpGenes to determine which features (probes/bins) are related to which genes
bpStats to determine which gene breaks are recurrent in the cohort

---

geneChromosomes,CopyNumberBreakPointGenes-method
Access Object geneChromosomes. This method returns a vector with gene chromosomes.

Description

Access Object geneChromosomes. This method returns a vector with gene chromosomes.

Usage

```
## S4 method for signature 'CopyNumberBreakPointGenes'
geneChromosomes(object)
```

Arguments

object An object of class CopyNumberBreakPoints

Value

vector with gene chromosomes

Examples

```
data( copynumber.data.chr20 )
data( ens.gene.ann.hg18 )
bp <- getBreakpoints( copynumber.data.chr20 )
bp <- bpFilter( bp )
bp <- addGeneAnnotation( bp, ens.gene.ann.hg18 )
bp <- bpGenes( bp )

geneChromosomes( bp )
```

---

geneInfo,CopyNumberBreakPointGenes-method

Access Options geneInfo. This method returns a dataframe with gene annotations.

Description

Access Options geneInfo. This method returns a dataframe with gene annotations.

Usage

```
## S4 method for signature 'CopyNumberBreakPointGenes'
geneInfo(object)
```

Arguments

object of class CopyNumberBreakPointGenes

Value

data.frame with gene annotations

Examples

```
data( copynumber.data.chr20 )
data( ens.gene.ann.hg18 )
bp <- getBreakpoints( copynumber.data.chr20 )
bp <- bpFilter( bp )
bp <- addGeneAnnotation( bp, ens.gene.ann.hg18 )

geneInfo( bp )
```

`getBreakpoints` *getBreakpoints*

Description

Builds the CopyNumberBreakPoints object from copynumber data and detects breakpoint locations.

Usage

```
getBreakpoints(data, data2 = NULL, first.rm = TRUE)
```

Arguments

`data`	An object of class **cghCall** or an object of class QDNAseqCopyNumbers or a data.frame containing feature annotations ("Chromosome", "Start", "End", "FeatureName") followed by copy number segment values (rows are features, columns are subjects).
`data2`	A "data.frame" containing copy number calls following feature annotations with the four columns ("Chromosome", "Start", "End", "FeatureName", ...). This is optional and allows CNA-associated breakpoint filtering. (see ?bpFilter)
`first.rm`	Remove the first 'artificial' breakpoint of the first DNA segment for each chromosome (default: first.rm=TRUE)

Details

The accuracy of chromosomal breakpoint locations depends on the quality and genomic resolution of processed copy number data. For CNA input data, we recommend to use established computational methods for CNA detection such as 'CGHcall' (Van De Wiel et al., 2007) for array-CGH or 'QDNAseq' (Scheinin et al., 2014) for MPS data, which both use the implemented Circular Binary Segmentation algorithm (Olshen et al. 2004).

Value

Returns an object of class CopyNumberBreakPoints.

References

Van De Wiel, M.A. et al. (2007) CGHcall: calling aberrations for array CGH tumor profiles. Bioinformatics, 23, 892-894.

Scheinin, I. et al. (2014) DNA copy number analysis of fresh and formalin-fixed specimens by shallow whole-genome sequencing with identification and exclusion of problematic regions in the genome assembly. Genome Research, 24, 2022-2032.

Olshen, A.B. et al. (2004) Circular binary segmentation for the analysis of array-based DNA copy number data. 5, 557-572.

Examples

```
data( copynumber.data.chr20 )
breakpoints <- getBreakpoints( data = copynumber.data.chr20 )

## or alternatively
library(CGHcall)
cgh <- copynumber.data.chr20
segmented <- data.frame( Chromosome=chromosomes(cgh), Start=bpstart(cgh),
  End=bpend(cgh), FeatureName=rownames(cgh), segmented(cgh))
called <- data.frame( Chromosome=chromosomes(cgh), Start=bpstart(cgh),
  End=bpend(cgh), FeatureName=rownames(cgh), calls(cgh))
breakpoints <- getBreakpoints( data = segmented, data2 = called )

## options to inspect the data
breakpoints
accessOptions( breakpoints )
```

---

namesFeatures,CopyNumberBreakPoints-method

Access Object namesFeatures. This method returns a vector with feature names.

Description

Access Object namesFeatures. This method returns a vector with feature names.

Usage

```
## S4 method for signature 'CopyNumberBreakPoints'
namesFeatures(object)
```

Arguments

object	An object of class CopyNumberBreakPoints

Value

a vector with feature names

Examples

```
data( copynumber.data.chr20 )
bp <- getBreakpoints( copynumber.data.chr20 )

namesFeatures( bp )
```

---

recurrentGenes,CopyNumberBreakPointGenes-method
Access Options recurrentGenes. This method returns a dataframe that contains genes that are recurrently affected across samples based on a FDR threshold.

Description

Access Options recurrentGenes. This method returns a dataframe that contains genes that are recurrently affected across samples based on a FDR threshold.

Usage

```
## S4 method for signature 'CopyNumberBreakPointGenes'
recurrentGenes(object,
  fdr.threshold = 0.1, summarize = TRUE, order.column = "FDR")
```

Arguments

`object`	Output of bpStats(): an object of class `CopyNumberBreakPointGenes`
`fdr.threshold`	A numeric Genes with lower FDR are returned
`summarize`	A logical to determine whether to only output a selection of columns
`order.column`	Name of the column to sort output on

Value

data.frame with genes recurrently affected by breakpoints

Examples

```
data( copynumber.data.chr20 )
data( ens.gene.ann.hg18 )
bp <- getBreakpoints( copynumber.data.chr20 )
bp <- bpFilter( bp )
bp <- addGeneAnnotation( bp, ens.gene.ann.hg18 )
bp <- bpGenes( bp )
bp <- bpStats( bp )

recurrentGenes( bp )
```

---

sampleNames,CopyNumberBreakPoints-method
Access Object sampleNames. This method returns a vector with sample names.

Description

Access Object sampleNames. This method returns a vector with sample names.

Usage

```
## S4 method for signature 'CopyNumberBreakPoints'
sampleNames(object)
```

Arguments

object	An object of class CopyNumberBreakPoints

Value

a vector with sample names

Examples

```
data( copynumber.data.chr20 )
bp <- getBreakpoints( copynumber.data.chr20 )

sampleNames( bp )
```

---

segmentData,CopyNumberBreakPoints-method

Access Object segmentData. This method returns a dataframe with segment values.

Description

Access Object segmentData. This method returns a dataframe with segment values.

Usage

```
## S4 method for signature 'CopyNumberBreakPoints'
segmentData(object)
```

Arguments

object	An object of class CopyNumberBreakPoints

Value

a dataframe with segment values

Examples

```
data( copynumber.data.chr20 )
bp <- getBreakpoints( copynumber.data.chr20 )

segmentData( bp )
```

Index

∗Topic **datasets**
copynumber.data.chr18, 9
copynumber.data.chr20, 10
copynumber.data.chr21, 10
ens.gene.ann.hg18, 13
ens.gene.ann.hg19, 14
ens.gene.ann.hg38, 14

accessOptions
(accessOptions,CopyNumberBreakPoints-method), 2
accessOptions,CopyNumberBreakPoints-method, 2
addGeneAnnotation, *17*
addGeneAnnotation
(addGeneAnnotation,CopyNumberBreakPoints-method), 3
addGeneAnnotation,CopyNumberBreakPoints-method, 3

bpFilter, *17*
bpFilter
(bpFilter,CopyNumberBreakPoints-method), 4
bpFilter,CopyNumberBreakPoints-method, 4
bpGenes, *17*
bpGenes
(bpGenes,CopyNumberBreakPointGenes-method), 5
bpGenes,CopyNumberBreakPointGenes-method, 5
bpPlot
(bpPlot,CopyNumberBreakPoints-method), 5
bpPlot,CopyNumberBreakPoints-method, 5
bpStats, *17*
bpStats
(bpStats,CopyNumberBreakPoints-method), 6
bpStats,CopyNumberBreakPoints-method, 6
breakpointData
(breakpointData,CopyNumberBreakPoints-method), 8
breakpointData,CopyNumberBreakPoints-method, 8
breakpointsPerGene
(breakpointsPerGene,CopyNumberBreakPointGenes-m
8
breakpointsPerGene,CopyNumberBreakPointGenes-method, 8

callBins, *17*
callData
(callData,CopyNumberBreakPoints-method), 9
callData,CopyNumberBreakPoints-method, 9
CGHcall, *17*
copynumber.data.chr18, 9
copynumber.data.chr20, 10
copynumber.data.chr21, 10
CopyNumberBreakPointGenes, *3*, *5–7*
CopyNumberBreakPointGenes
(CopyNumberBreakPointGenes-class), 11
CopyNumberBreakPointGenes-class, 11
CopyNumberBreakPoints, *3*, *4*, *6*, *11*, *12*, *19*
CopyNumberBreakPoints
(CopyNumberBreakPoints-class), 12
CopyNumberBreakPoints-class, 12

ens.gene.ann.hg18, 13
ens.gene.ann.hg19, 14
ens.gene.ann.hg38, 14

featureChromosomes
(featureChromosomes,CopyNumberBreakPoints-metho
15
featureChromosomes,CopyNumberBreakPoints-method, 15
featureInfo
(featureInfo,CopyNumberBreakPoints-method), 16
featureInfo,CopyNumberBreakPoints-method, 16

featuresPerGene
(featuresPerGene,CopyNumberBreakPointGenes-method),
16
featuresPerGene,CopyNumberBreakPointGenes-method,
16

GeneBreak, 17
GeneBreak-package (GeneBreak), 17
geneChromosomes
(geneChromosomes,CopyNumberBreakPointGenes-method),
17
geneChromosomes,CopyNumberBreakPointGenes-method,
17
geneInfo
(geneInfo,CopyNumberBreakPointGenes-method),
18
geneInfo,CopyNumberBreakPointGenes-method,
18
getBreakpoints, *17*, 19

namesFeatures
(namesFeatures,CopyNumberBreakPoints-method),
20
namesFeatures,CopyNumberBreakPoints-method,
20

recurrentGenes
(recurrentGenes,CopyNumberBreakPointGenes-method),
21
recurrentGenes,CopyNumberBreakPointGenes-method,
21

sampleNames
(sampleNames,CopyNumberBreakPoints-method),
21
sampleNames,CopyNumberBreakPoints-method,
21
segmentData
(segmentData,CopyNumberBreakPoints-method),
22
segmentData,CopyNumberBreakPoints-method,
22
