# Supplementary material for: GeneBreak: detection of recurrent DNA copy number aberration-associated chromosomal breakpoints within genes
Source: F1000Res. 2017 Jul 6;5:2340. Originally published 2016 Sep 19. [Version 2] doi: 10.12688/f1000research.9259.2 (PMC5500957; doi:10.12688/f1000research.9259.2)
Supplement: Peer review history

# Peer review: Referee response for version 2

**DOI:** 10.5256/f1000research.12600.r24055

**Published:** 2017-07-06

**Authors:** Angel Rubio

**Affiliations:** 1 Group of Bioinformatics, TECNUN, University of Navarra, San Sebastian, Spain

The authors provided an brief explanation of the statistics involved in the detection of recurrent copy number changes. I would have liked a slightly deeper description but, for most users the explanation is sufficient and give an idea of the methodology. 

 I think that this paper is ready.

I have read this submission. I believe that I have an appropriate level of expertise to confirm that it is of an acceptable scientific standard.

# Peer review: Referee response for version 1

**DOI:** 10.5256/f1000research.9967.r18598

**Published:** 2017-02-13

**Authors:** Angel Rubio

**Affiliations:** 1 Group of Bioinformatics, TECNUN, University of Navarra, San Sebastian, Spain

The paper shows an inspiring vision of the copy number changes in the genome focusing on the "changes" more than on the levels of change. The underlying reasoning is that a copy number change, if occurs within the loci occupied by a gene, implies an alteration in the coding sequence of the gene.

 In addition, it is shown that these changes occur recurrently, i.e. the loci where the copy number changes tend to be similar in different samples with the same type of cancer.

 The methodology has been uploaded to Bioconductor. The stringent quality checks of Bioconductor guarantees the availability for different platforms and, in fact, the vignette is easy to follow and use.

 My main concern with this paper is the (lack of) description of the statistical method to state the recurrence of the copy number changes. Within the methods section is only stated that there are two methods (genome location and gene-level) but the differences between them or the underlying statistical model is missing.

I have read this submission. I believe that I have an appropriate level of expertise to confirm that it is of an acceptable scientific standard, however I have significant reservations, as outlined above.

# Peer review: Referee response for version 1

**DOI:** 10.5256/f1000research.9967.r16416

**Published:** 2017-01-09

**Authors:** Tobias Marschall

**Affiliations:** 1 Center for Bioinformatics, Max-Planck Institute for Infomatics, Saarbrücken, Germany

GeneBreak is an R package to help identifying recurrent breakpoints of copy number variants (CNVs). While the offered analyses are straightforward from a methodological point of view, this package can be valuable in practice, providing an easy and reproducible way to conduct such analyses. I appreciate that it is available from bioconductor (and hence easily installable), openly developed on github, and archived on zenodo.

 I merely have some minor suggestions for improvements:

- When trying to use the package, I couldn't open the example data (got a "data set ‘copynumber.data.chr20’ not found" error). Could you verify that it's available?
- First sentence: SNV commonly means "single nucleotide variation" (not "small").
- P1, L9: " *Recently, ...*" Of course what you consider "recent" is a matter of taste, but here you are citing a review paper from 2007. I wouldn't call this recent.
- P1, Methods, L3: " *... and copy number detection algorithm*" Either explain what exactly you mean here, or remove.
- P1, paragraph " *Due to the typical granularity [...], in fact represent a chromosomal interval.*" I can guess what you mean here, but writing this more clearly would be good.
- P1, " *This method assumes the same permutation null- distribution for all candidate breakpoint events for the analysis of breakpoints at the level of genomic location.*" Could you describe in more detail how the null distribution is obtained? 
- P1, " *In addition, a more comprehensive and powerful dedicated Benjamini-Hochberg FDR correction that accounts for discreteness in the null-distribution is supplied.*" The Benjamini-Hochberg procedure is a well defined statistical method. I would rephrase the respective sentence(s) to explicitly say that you are talking about Gilbert's method.

I have read this submission. I believe that I have an appropriate level of expertise to confirm that it is of an acceptable scientific standard.
